# Supplementary material for: Severe Acute Kidney Injury in Cardiovascular Surgery: Thrombotic Microangiopathy as a Differential Diagnosis to Ischemia Reperfusion Injury. A Retrospective Study
Source: J Clin Med. 2020 Sep 8;9(9):2900. doi: 10.3390/jcm9092900 (PMC7565159; doi:10.3390/jcm9092900)
Supplement: Supplementary file 1 [file jcm-09-02900-s001.zip › jcm-914727-supplementary.docx]

**Supplementary Table 1.** Postoperative laboratory parameters (day 0 – 7).

|  | **TMA**  **(*n* = 15)** | | **Non-TMA**  **(*n* = 54)** | | ***p*-value** |
| --- | --- | --- | --- | --- | --- |
|  | Median (IQR) | | Median (IQR) | |  |
| Bilirubin (mg/dl) (max.) | 3.6 | (2.9 – 5.7) | 3.6 | (2.2 – 6.9) | 0.699 |
| LDH (U/l) (max.) | 1777 | (859 – 2631) | 891 | (560 – 2890) | 0.103 |
| Hemoglobin (g/dl) (min.) | 7.8 | (7.2 – 8) | 8.9 | (7.6 – 8.5) | 0.149 |
| Thrombocytes (G/l) (min.) | 40 | (28 – 45) | 55 | (37 – 73) | 0.004 |
| INR (max.) | 1.2 | (1.1 – 1.5) | 1.35 | (1.2 – 1.9) | 0.064 |
| Fibrinogen (mg/dl) (min.) | 419 | (338 – 590) | 414 | (267 – 558) | 0.477 |
| D-Dimer (µg/dl) (max.) | 8 | (5.4 – 12.2) | 9.8 | (3.5 – 17.9) | 0.793 |

**Supplementary Table 1.** LDH: lactate dehydrogenase, INR: international normalized ratio, min.: minimal value day 0-7, max.: maximal value day 0-7.

**Supplementary Table 2.** Kidney function parameters on discharge.

|  | **TMA**  **(*n* = 15)**  Median (IQR) | | **Non-TMA**  **(*n* = 54)**  Median (IQR*)* | | ***p*-value** |
| --- | --- | --- | --- | --- | --- |
|  |  |  |  |  |  |
| Creatinine (mg/dl) | 2.3 | (1.0 – 4.0) | 1.7 | (1.3 – 2.4) | 0.322 |
| eGFR (ml/min) | 32 | (0 – 66) | 19 | (0 – 48) | 0.286 |
| Urea (mg/dl) | 80 | (40 – 111) | 75 | (41 – 98) | 0.824 |
| Discharged on dialysis n, (%) | 4 | (26.7) | 10 | (18.5) | 0.491 |
| Time on dialysis (days) | 20 | (14 – 30) | 7.5 | (2 – 25) | **0.019** |
| Δ eGFR (ml/min) | −54 | (−8 – −81) | −33.5 | (−12 – −59) | 0.265 |
|  |  |  |  |  |  |
| **Survivors only** | (***n* = 15)**  Median (IQR) | | **(*n* = 30)**  Median (IQR*)* | | ***p*-value** |
| Creatinine (mg/dl) | 2.3 | (1.0 – 4.0) | 1.55 | (1.2 – 2.7) | 0.347 |
| eGFR (ml/min) | 32 | (0 – 66) | 45 | (0 – 67) | 0.743 |
| Urea (mg/dl) | 80 | (40 – 111) | 78 | (35 – 108) | 0.885 |
| Discharged on dialysis (n, %) | 4 | (26.7) | 10 | (33.3) | 0.743 |
| Time on dialysis (days) | 20 | (14 – 30) | 20 | (4 – 30) | 0.365 |
| Δ eGFR (ml/min) | −54 | (−8 – −81) | −17 | (+2 – −80) | 0.062 |
|  |  |  |  |  |  |

**Supplementary Table 2.** eGFR: estimated glomerular filtration rate calculated with CKD-EPI formula, Δ eGFR = (eGFR at discharge) – (eGFR at baseline), w/o ESKD: without ESKD, CVS: cardiovascular surgery.

**Supplementary Table 3.** In-hospital length of stay.

|  | **TMA**  **(*n* = 15)**  Median (IQR) | | **Non-TMA**  **(*n* = 54)**  Median (IQR) | | ***p*-value** | |
| --- | --- | --- | --- | --- | --- | --- |
|  |  |  |  |  |  |  |
|  |  |  |  |  |  |  |
| Mechanical ventilation (d) | 4 | (2 – 6.5) | 7 | (3 – 20) | | 0.019 |
| ICU (d) | 12 | (10 - 27) | 17 | (7 – 33) | | 0.575 |
| Discharge (d) | 28 | (23 – 39) | 22 | (9 – 48) | | 0.238 |
|  |  |  |  |  | | |

**Supplementary Table 3.** d: days.

**Supplementary Figure 1.** In-hospital evolution of kidney function (baseline vs discharge).


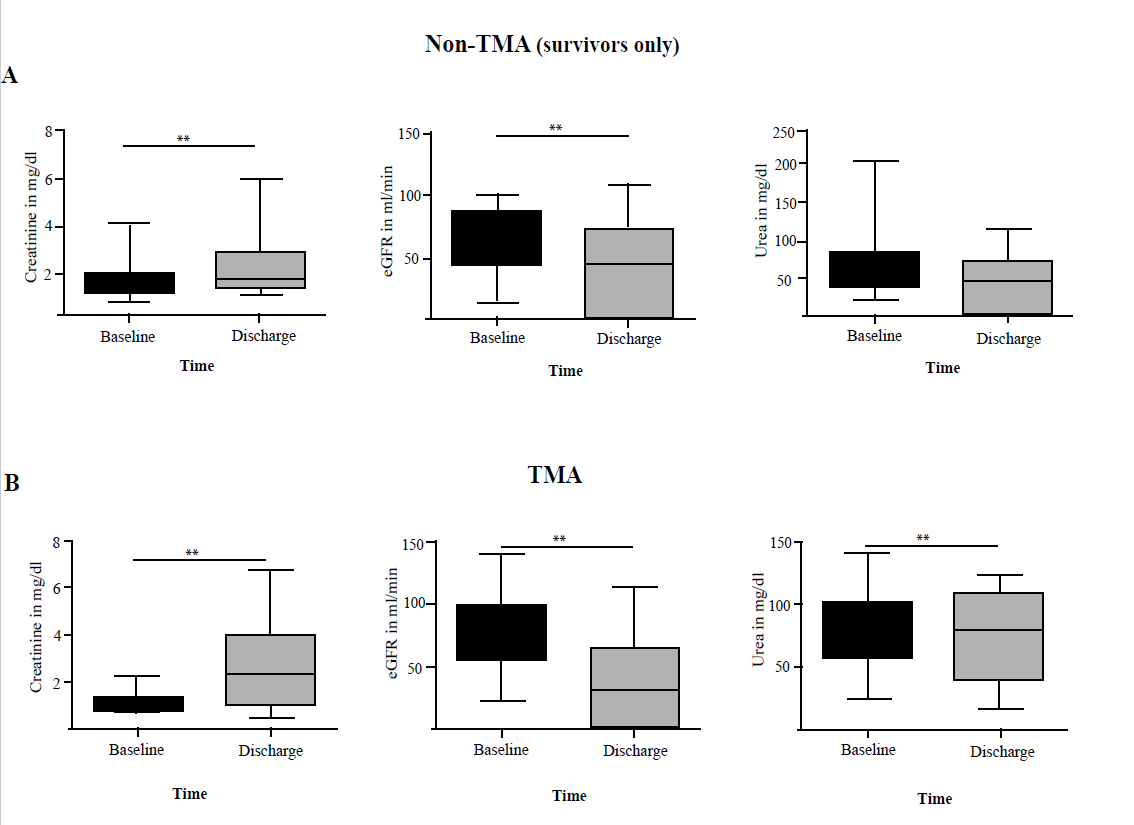


**Supplementary Figure 1.** In-hospital evolution of kidney function (baseline vs discharge). **A:** Surrogate parameters of kidney function in non-TMA patients (survivors only). **B:** Surrogate parameters of kidney function in TMA patients. eGFR: estimated glomerular filtration rate. ** = p<0.01
